# Supplementary material for: Permutation-based methods for mediation analysis in studies with small sample sizes
Source: PeerJ. 2020 Jan 22;8:e8246. doi: 10.7717/peerj.8246 (PMC6982415; doi:10.7717/peerj.8246)
Supplement: Supplemental Information 1 [file peerj-08-8246-s001.docx]

**Online Supplemental Material**

Table S1. Null Conditions Evaluated

| $\boldsymbol{\rho}_{\boldsymbol{XC}}$ | $\boldsymbol{\rho}_{\boldsymbol{XM}}$ | $\boldsymbol{\rho}_{\boldsymbol{CM}}$ | $\boldsymbol{\rho}_{\boldsymbol{XY}}$ | $\boldsymbol{\rho}_{\boldsymbol{CY}}$ | $\boldsymbol{\rho}_{\boldsymbol{MY}}$ | $\boldsymbol{\alpha}_{\boldsymbol{1}}$ | $\boldsymbol{\alpha}_{\boldsymbol{2}}$ | $\boldsymbol{\gamma}_{\boldsymbol{1}}$ | $\boldsymbol{\gamma}_{\boldsymbol{2}}$ | $\boldsymbol{\gamma}_{\boldsymbol{3}}$ |
| --- | --- | --- | --- | --- | --- | --- | --- | --- | --- | --- |
| C as a confounder between X and Y | | | | | | | | | | |
| $\alpha_{1}=0$, $\gamma_{3}=0$ | | | | | | | | | | |
| 0.15 | 0 | 0 | 0.15 | 0.15 | 0 | 0 | 0 | 0.1304 | 0.1304 | 0 |
| 0.6 | 0 | 0 | 0.6 | 0.6 | 0 | 0 | 0 | 0.375 | 0.375 | 0 |
| $\alpha_{1}=0$, $\gamma_{3}\neq0$ | | | | | | | | | | |
| 0.15 | 0 | 0 | 0.15 | 0.15 | 0.15 | 0 | 0 | 0.1304 | 0.1304 | 0.15 |
| 0.15 | 0 | 0 | 0.15 | 0.15 | 0.3 | 0 | 0 | 0.1304 | 0.1304 | 0.3 |
| 0.15 | 0 | 0 | 0.15 | 0.15 | 0.6 | 0 | 0 | 0.1304 | 0.1304 | 0.6 |
| 0.6 | 0 | 0 | 0.6 | 0.6 | 0.15 | 0 | 0 | 0.375 | 0.375 | 0.15 |
| 0.6 | 0 | 0 | 0.6 | 0.6 | 0.3 | 0 | 0 | 0.375 | 0.375 | 0.3 |
| 0.6 | 0 | 0 | 0.6 | 0.6 | 0.6 | 0 | 0 | 0.375 | 0.375 | 0.6 |
| C as a covariate | | | | | | | | | | |
| $\alpha_{1}=0$, $\gamma_{3}=0$ | | | | | | | | | | |
| 0 | 0 | 0 | 0.15 | 0.15 | 0 | 0 | 0 | 0.15 | 0.15 | 0 |
| 0 | 0 | 0 | 0.6 | 0.6 | 0 | 0 | 0 | 0.6 | 0.6 | 0 |
| $\alpha_{1}=0$, $\gamma_{3}\neq0$ | | | | | | | | | | |
| 0 | 0 | 0 | 0.15 | 0.15 | 0.15 | 0 | 0 | 0.15 | 0.15 | 0.15 |
| 0 | 0 | 0 | 0.15 | 0.15 | 0.3 | 0 | 0 | 0.15 | 0.15 | 0.3 |
| 0 | 0 | 0 | 0.15 | 0.15 | 0.6 | 0 | 0 | 0.15 | 0.15 | 0.6 |
| 0 | 0 | 0 | 0.6 | 0.6 | 0.15 | 0 | 0 | 0.6 | 0.6 | 0.15 |
| 0 | 0 | 0 | 0.6 | 0.6 | 0.3 | 0 | 0 | 0.6 | 0.6 | 0.3 |
| $\alpha_{1}\neq0$, $\gamma_{3}=0$ | | | | | | | | | | |
| 0 | 0.15 | 0 | 0 | 0.15 | 0 | 0.15 | 0 | 0 | 0.15 | 0 |
| 0 | 0.3 | 0 | 0 | 0.15 | 0 | 0.3 | 0 | 0 | 0.15 | 0 |
| 0 | 0.6 | 0 | 0 | 0.15 | 0 | 0.6 | 0 | 0 | 0.15 | 0 |
| 0 | 0.15 | 0 | 0 | 0.6 | 0 | 0.15 | 0 | 0 | 0.6 | 0 |
| 0 | 0.3 | 0 | 0 | 0.6 | 0 | 0.3 | 0 | 0 | 0.6 | 0 |
| 0 | 0.6 | 0 | 0 | 0.6 | 0 | 0.6 | 0 | 0 | 0.6 | 0 |
| C as a confounder between M and Y | | | | | | | | | | |
| $\alpha_{1}=0$, $\gamma_{3}\neq0$ | | | | | | | | | | |
| 0 | 0 | 0.15 | 0.15 | 0.15 | 0.15 | 0 | 0.15 | 0.15 | 0.1304 | 0.1304 |
| 0 | 0 | 0.15 | 0.15 | 0.15 | 0.3 | 0 | 0.15 | 0.15 | 0.1074 | 0.2839 |
| 0 | 0 | 0.15 | 0.15 | 0.15 | 0.6 | 0 | 0.15 | 0.15 | 0.0614 | 0.5908 |
| 0 | 0 | 0.6 | 0.6 | 0.6 | 0.15 | 0 | 0.6 | 0.6 | 0.7969 | -0.3281 |
| 0 | 0 | 0.6 | 0.6 | 0.6 | 0.3 | 0 | 0.6 | 0.6 | 0.6563 | -0.0938 |
| 0 | 0 | 0.6 | 0.6 | 0.6 | 0.6 | 0 | 0.6 | 0.6 | 0.375 | 0.375 |

Table S2. Alternative Conditions Evaluated

| $\boldsymbol{\rho}_{\boldsymbol{XC}}$ | $\boldsymbol{\rho}_{\boldsymbol{XM}}$ | $\boldsymbol{\rho}_{\boldsymbol{CM}}$ | $\boldsymbol{\rho}_{\boldsymbol{XY}}$ | $\boldsymbol{\rho}_{\boldsymbol{CY}}$ | $\boldsymbol{\rho}_{\boldsymbol{MY}}$ | $\boldsymbol{\alpha}_{\boldsymbol{1}}$ | $\boldsymbol{\alpha}_{\boldsymbol{2}}$ | $\boldsymbol{\gamma}_{\boldsymbol{1}}$ | $\boldsymbol{\gamma}_{\boldsymbol{2}}$ | $\boldsymbol{\gamma}_{\boldsymbol{3}}$ |
| --- | --- | --- | --- | --- | --- | --- | --- | --- | --- | --- |
| C as a confounder between X and Y | | | | | | | | | | |
| 0.15 | 0.15 | 0 | 0.15 | 0.15 | 0.15 | 0.1535 | -0.0230 | 0.1099 | 0.1335 | 0.1335 |
| 0.15 | 0.15 | 0 | 0.15 | 0.15 | 0.3 | 0.1535 | -0.0230 | 0.0864 | 0.1370 | 0.2870 |
| 0.15 | 0.15 | 0 | 0.15 | 0.15 | 0.6 | 0.1535 | -0.0230 | 0.0393 | 0.1441 | 0.5941 |
| 0.15 | 0.3 | 0 | 0.15 | 0.15 | 0.15 | 0.3069 | -0.0460 | 0.0930 | 0.1361 | 0.1221 |
| 0.15 | 0.3 | 0 | 0.15 | 0.15 | 0.3 | 0.3069 | -0.0460 | 0.0423 | 0.1437 | 0.2873 |
| 0.15 | 0.3 | 0 | 0.15 | 0.15 | 0.6 | 0.3069 | -0.0460 | -0.0592 | 0.1589 | 0.6177 |
| 0.15 | 0.6 | 0 | 0.15 | 0.15 | 0.15 | 0.6138 | -0.0921 | 0.0607 | 0.1409 | 0.1136 |
| 0.15 | 0.6 | 0 | 0.15 | 0.15 | 0.3 | 0.6138 | -0.0921 | -0.0850 | 0.1628 | 0.3510 |
| 0.15 | 0.6 | 0 | 0.15 | 0.15 | 0.6 | 0.6138 | -0.0921 | -0.3765 | 0.2065 | 0.8259 |
| 0.6 | 0.15 | 0 | 0.6 | 0.6 | 0.15 | 0.2344 | -0.1406 | 0.3522 | 0.3887 | 0.0972 |
| 0.6 | 0.15 | 0 | 0.6 | 0.6 | 0.3 | 0.2344 | -0.1406 | 0.3158 | 0.4105 | 0.2526 |
| 0.6 | 0.15 | 0 | 0.6 | 0.6 | 0.6 | 0.2344 | -0.1406 | 0.2429 | 0.4543 | 0.5636 |
| 0.6 | 0.3 | 0 | 0.6 | 0.6 | 0.15 | 0.4688 | -0.2813 | 0.3545 | 0.3873 | 0.0436 |
| 0.6 | 0.3 | 0 | 0.6 | 0.6 | 0.3 | 0.4688 | -0.2813 | 0.2727 | 0.4364 | 0.2182 |
| 0.6 | 0.3 | 0 | 0.6 | 0.6 | 0.6 | 0.4688 | -0.2813 | 0.1091 | 0.5345 | 0.5673 |
| 0.6 | 0.6 | 0 | 0.6 | 0.6 | 0.15 | 0.9375 | -0.5625 | 0.5357 | 0.2786 | 0.1714 |
| 0.6 | 0.6 | 0 | 0.6 | 0.6 | 0.3 | 0.9375 | -0.5625 | 0.2143 | 0.4714 | 0.1714 |
| 0.6 | 0.6 | 0 | 0.6 | 0.6 | 0.6 | 0.9375 | -0.5625 | -0.4286 | 0.8571 | 0.8571 |
| C as a confounder between M and Y | | | | | | | | | | |
| 0 | 0.15 | 0.15 | 0.15 | 0.15 | 0.15 | 0.15 | 0.15 | 0.1335 | 0.1335 | 0.1099 |
| 0 | 0.15 | 0.15 | 0.15 | 0.15 | 0.3 | 0.15 | 0.15 | 0.1099 | 0.1099 | 0.2670 |
| 0 | 0.15 | 0.15 | 0.15 | 0.15 | 0.6 | 0.15 | 0.15 | 0.0628 | 0.0628 | 0.5812 |
| 0 | 0.3 | 0.15 | 0.15 | 0.15 | 0.15 | 0.3 | 0.15 | 0.1221 | 0.1361 | 0.0930 |
| 0 | 0.3 | 0.15 | 0.15 | 0.15 | 0.3 | 0.3 | 0.15 | 0.0714 | 0.1107 | 0.2620 |
| 0 | 0.3 | 0.15 | 0.15 | 0.15 | 0.6 | 0.3 | 0.15 | -0.0300 | 0.0600 | 0.6000 |
| 0 | 0.6 | 0.15 | 0.15 | 0.15 | 0.15 | 0.6 | 0.15 | 0.1136 | 0.1409 | 0.0607 |
| 0 | 0.6 | 0.15 | 0.15 | 0.15 | 0.3 | 0.6 | 0.15 | -0.0322 | 0.1045 | 0.3036 |
| 0 | 0.6 | 0.15 | 0.15 | 0.15 | 0.6 | 0.6 | 0.15 | -0.3237 | 0.0316 | 0.7895 |
| 0 | 0.15 | 0.6 | 0.6 | 0.6 | 0.15 | 0.15 | 0.6 | 0.6729 | 0.8915 | -0.4858 |
| 0 | 0.15 | 0.6 | 0.6 | 0.6 | 0.3 | 0.15 | 0.6 | 0.6364 | 0.7457 | -0.2429 |
| 0 | 0.15 | 0.6 | 0.6 | 0.6 | 0.6 | 0.15 | 0.6 | 0.5636 | 0.4543 | 0.2429 |
| 0 | 0.3 | 0.6 | 0.6 | 0.6 | 0.15 | 0.3 | 0.6 | 0.8127 | 1.0255 | -0.7091 |
| 0 | 0.3 | 0.6 | 0.6 | 0.6 | 0.3 | 0.3 | 0.6 | 0.7309 | 0.8618 | -0.4364 |
| 0 | 0.3 | 0.6 | 0.6 | 0.6 | 0.6 | 0.3 | 0.6 | 0.5673 | 0.5345 | 0.1091 |
| C as a covariate | | | | | | | | | | |
| 0 | 0.15 | 0 | 0.15 | 0.15 | 0.15 | 0.15 | 0 | 0.1304 | 0.15 | 0.1304 |
| 0 | 0.15 | 0 | 0.15 | 0.15 | 0.3 | 0.15 | 0 | 0.1074 | 0.15 | 0.2839 |
| 0 | 0.15 | 0 | 0.15 | 0.15 | 0.6 | 0.15 | 0 | 0.0614 | 0.15 | 0.5908 |
| 0 | 0.3 | 0 | 0.15 | 0.15 | 0.15 | 0.3 | 0 | 0.1154 | 0.15 | 0.1154 |
| 0 | 0.3 | 0 | 0.15 | 0.15 | 0.3 | 0.3 | 0 | 0.0659 | 0.15 | 0.2802 |
| 0 | 0.3 | 0 | 0.15 | 0.15 | 0.6 | 0.3 | 0 | -0.0330 | 0.15 | 0.6099 |
| 0 | 0.6 | 0 | 0.15 | 0.15 | 0.15 | 0.6 | 0 | 0.0938 | 0.15 | 0.0938 |
| 0 | 0.6 | 0 | 0.15 | 0.15 | 0.3 | 0.6 | 0 | -0.0469 | 0.15 | 0.3281 |
| 0 | 0.6 | 0 | 0.15 | 0.15 | 0.6 | 0.6 | 0 | -0.3281 | 0.15 | 0.7969 |
| 0 | 0.15 | 0 | 0.6 | 0.6 | 0.15 | 0.15 | 0 | 0.5908 | 0.6 | 0.0614 |
| 0 | 0.15 | 0 | 0.6 | 0.6 | 0.3 | 0.15 | 0 | 0.5678 | 0.6 | 0.2148 |
| 0 | 0.15 | 0 | 0.6 | 0.6 | 0.6 | 0.15 | 0 | 0.5217 | 0.6 | 0.5217 |
| 0 | 0.3 | 0 | 0.6 | 0.6 | 0.15 | 0.3 | 0 | 0.6099 | 0.6 | -0.0330 |
| 0 | 0.3 | 0 | 0.6 | 0.6 | 0.3 | 0.3 | 0 | 0.5604 | 0.6 | 0.1319 |
| 0 | 0.3 | 0 | 0.6 | 0.6 | 0.6 | 0.3 | 0 | 0.4615 | 0.6 | 0.4615 |
| 0 | 0.6 | 0 | 0.6 | 0.6 | 0.15 | 0.6 | 0 | 0.7969 | 0.6 | -0.3281 |
| 0 | 0.6 | 0 | 0.6 | 0.6 | 0.3 | 0.6 | 0 | 0.6563 | 0.6 | -0.0938 |
| 0 | 0.6 | 0 | 0.6 | 0.6 | 0.6 | 0.6 | 0 | 0.3750 | 0.6 | 0.3750 |

Table S3. Results from Null Conditions, Type I error for C as a covariate

| **Conditions** | | | | | | | | **Type I error rates** | | |
| --- | --- | --- | --- | --- | --- | --- | --- | --- | --- | --- |
| $\boldsymbol{\rho}_{\boldsymbol{XC}}$ | $\boldsymbol{\rho}_{\boldsymbol{XM}}$ | $\boldsymbol{\rho}_{\boldsymbol{CM}}$ | $\boldsymbol{\rho}_{\boldsymbol{XY}}$ | $\boldsymbol{\rho}_{\boldsymbol{CY}}$ | $\boldsymbol{\rho}_{\boldsymbol{MY}}$ | $\boldsymbol{\alpha}_{\boldsymbol{1}}$ | $\boldsymbol{\gamma}_{\boldsymbol{3}}$ | **IERM** | **PSRM** | **IEFM** |
| n=30 | | | | | | | | | | |
| $\alpha_{1}=0$, $\gamma_{3}=0$ | | | | | | | | | | |
| 0 | 0 | 0 | 0.15 | 0.15 | 0 | 0 | 0 | 0.055 | 0.002 | 0.003 |
| 0 | 0 | 0 | 0.6 | 0.6 | 0 | 0 | 0 | **0.064** | 0.005 | 0.006 |
| $\alpha_{1}=0$, $\gamma_{3}\neq0$ | | | | | | | | | | |
| 0 | 0 | 0 | 0.15 | 0.15 | 0.15 | 0 | 0.15 | **0.098** | **0.011** | **0.011** |
| 0 | 0 | 0 | 0.15 | 0.15 | 0.3 | 0 | 0.3 | **0.214** | 0.024 | 0.030 |
| 0 | 0 | 0 | 0.15 | 0.15 | 0.6 | 0 | 0.6 | **0.496** | 0.040 | 0.061 |
| 0 | 0 | 0 | 0.6 | 0.6 | 0.15 | 0 | 0.15 | **0.198** | 0.012 | 0.015 |
| 0 | 0 | 0 | 0.6 | 0.6 | 0.3 | 0 | 0.3 | **0.470** | 0.064 | **0.080** |
| $\alpha_{1}\neq0$, $\gamma_{3}=0$ | | | | | | | | | | |
| 0 | 0.15 | 0 | 0 | 0.15 | 0 | 0.15 | 0 | **0.107** | 0.006 | 0.007 |
| 0 | 0.3 | 0 | 0 | 0.15 | 0 | 0.3 | 0 | **0.201** | 0.024 | 0.026 |
| 0 | 0.6 | 0 | 0 | 0.15 | 0 | 0.6 | 0 | **0.492** | 0.055 | **0.075** |
| 0 | 0.15 | 0 | 0 | 0.6 | 0 | 0.15 | 0 | **0.104** | 0.007 | 0.013 |
| 0 | 0.3 | 0 | 0 | 0.6 | 0 | 0.3 | 0 | **0.208** | 0.024 | 0.028 |
| 0 | 0.6 | 0 | 0 | 0.6 | 0 | 0.6 | 0 | **0.487** | 0.058 | **0.070** |
| n=100 | | | | | | | | | | |
| $\alpha_{1}=0$, $\gamma_{3}=0$ | | | | | | | | | | |
| 0 | 0 | 0 | 0.15 | 0.15 | 0 | 0 | 0 | 0.053 | 0 | 0 |
| 0 | 0 | 0 | 0.6 | 0.6 | 0 | 0 | 0 | 0.056 | 0 | 0 |
| $\alpha_{1}=0$, $\gamma_{3}\neq0$ | | | | | | | | | | |
| 0 | 0 | 0 | 0.15 | 0.15 | 0.15 | 0 | 0.15 | **0.188** | 0.015 | 0.009 |
| 0 | 0 | 0 | 0.15 | 0.15 | 0.3 | 0 | 0.3 | **0.461** | 0.045 | 0.041 |
| 0 | 0 | 0 | 0.15 | 0.15 | 0.6 | 0 | 0.6 | **0.694** | 0.049 | 0.052 |
| 0 | 0 | 0 | 0.6 | 0.6 | 0.15 | 0 | 0.15 | **0.402** | 0.042 | 0.041 |
| 0 | 0 | 0 | 0.6 | 0.6 | 0.3 | 0 | 0.3 | **0.683** | 0.045 | 0.050 |
| $\alpha_{1}\neq0$, $\gamma_{3}=0$ | | | | | | | | | | |
| 0 | 0.15 | 0 | 0 | 0.15 | 0 | 0.15 | 0 | **0.171** | 0.019 | 0.016 |
| 0 | 0.3 | 0 | 0 | 0.15 | 0 | 0.3 | 0 | **0.500** | 0.053 | 0.048 |
| 0 | 0.6 | 0 | 0 | 0.15 | 0 | 0.6 | 0 | **0.736** | 0.056 | 0.062 |
| 0 | 0.15 | 0 | 0 | 0.6 | 0 | 0.15 | 0 | **0.191** | 0.022 | 0.020 |
| 0 | 0.3 | 0 | 0 | 0.6 | 0 | 0.3 | 0 | **0.424** | 0.031 | 0.030 |
| 0 | 0.6 | 0 | 0 | 0.6 | 0 | 0.6 | 0 | **0.720** | 0.052 | 0.054 |

* Under 1000 simulations with a binary outcome of accept or reject, deviations in Type I error rates beyond a Wald confidence interval (0.036, 0.064) suggest deviations from a level 0.05 test not due to sampling. Scenarios where the error rates exceeded the Wald confidence bounds are bolded.

Table S4. Results from Null Conditions, Type I error for C as a confounder between M and Y

| **Conditions** | | | | | | | | **Type I error rates** | | |
| --- | --- | --- | --- | --- | --- | --- | --- | --- | --- | --- |
| $\boldsymbol{\rho}_{\boldsymbol{XC}}$ | $\boldsymbol{\rho}_{\boldsymbol{XM}}$ | $\boldsymbol{\rho}_{\boldsymbol{CM}}$ | $\boldsymbol{\rho}_{\boldsymbol{XY}}$ | $\boldsymbol{\rho}_{\boldsymbol{CY}}$ | $\boldsymbol{\rho}_{\boldsymbol{MY}}$ | $\boldsymbol{\alpha}_{\boldsymbol{1}}$ | $\boldsymbol{\gamma}_{\boldsymbol{3}}$ | **IERM** | **PSRM** | **IEFM** |
| n=30 | | | | | | | | | | |
| $\alpha_{1}=0$, $\gamma_{3}\neq0$ | | | | | | | | | | |
| 0 | 0 | 0.15 | 0.15 | 0.15 | 0.15 | 0 | 0.1304 | **0.077** | 0.004 | 0.005 |
| 0 | 0 | 0.15 | 0.15 | 0.15 | 0.3 | 0 | 0.2839 | **0.184** | 0.021 | 0.020 |
| 0 | 0 | 0.15 | 0.15 | 0.15 | 0.6 | 0 | 0.5908 | **0.502** | 0.050 | **0.064** |
| 0 | 0 | 0.6 | 0.6 | 0.6 | 0.15 | 0 | -0.3281 | **0.418** | 0.058 | **0.068** |
| 0 | 0 | 0.6 | 0.6 | 0.6 | 0.3 | 0 | -0.0938 | **0.080** | 0.009 | 0.010 |
| 0 | 0 | 0.6 | 0.6 | 0.6 | 0.6 | 0 | 0.375 | **0.465** | 0.052 | 0.062 |
| n=100 | | | | | | | | | | |
| $\alpha_{1}=0$, $\gamma_{3}\neq0$ | | | | | | | | | | |
| 0 | 0 | 0.15 | 0.15 | 0.15 | 0.15 | 0 | 0.1304 | **0.160** | 0.014 | 0.012 |
| 0 | 0 | 0.15 | 0.15 | 0.15 | 0.3 | 0 | 0.2839 | **0.417** | 0.047 | 0.044 |
| 0 | 0 | 0.15 | 0.15 | 0.15 | 0.6 | 0 | 0.5908 | **0.719** | 0.044 | 0.046 |
| 0 | 0 | 0.6 | 0.6 | 0.6 | 0.15 | 0 | -0.3281 | **0.667** | 0.046 | 0.052 |
| 0 | 0 | 0.6 | 0.6 | 0.6 | 0.3 | 0 | -0.0938 | **0.167** | 0.016 | 0.012 |
| 0 | 0 | 0.6 | 0.6 | 0.6 | 0.6 | 0 | 0.375 | **0.675** | 0.045 | 0.047 |

* Under 1000 simulations with a binary outcome of accept or reject, deviations in Type I error rates beyond a Wald confidence interval (0.036, 0.064) suggest deviations from a level 0.05 test not due to sampling. Scenarios where the error rates exceeded the Wald confidence bounds are bolded.

Table S5. Results from Alternative Conditions, Power for C as a Covariate

| **Conditions** | | | | | | | | **Power** | |
| --- | --- | --- | --- | --- | --- | --- | --- | --- | --- |
| $\boldsymbol{\rho}_{\boldsymbol{XC}}$ | $\boldsymbol{\rho}_{\boldsymbol{XM}}$ | $\boldsymbol{\rho}_{\boldsymbol{CM}}$ | $\boldsymbol{\rho}_{\boldsymbol{XY}}$ | $\boldsymbol{\rho}_{\boldsymbol{CY}}$ | $\boldsymbol{\rho}_{\boldsymbol{MY}}$ | $\boldsymbol{\alpha}_{\boldsymbol{1}}$ | $\boldsymbol{\gamma}_{\boldsymbol{3}}$ | **PSRM** | **IEFM** |
| n=30 | | | | | | | | | |
| 0 | 0.15 | 0 | 0.15 | 0.15 | 0.15 | 0.15 | 0.1304 | 0.011 | 0.015 |
| 0 | 0.15 | 0 | 0.15 | 0.15 | 0.3 | 0.15 | 0.2839 | 0.043 | 0.052 |
| 0 | 0.15 | 0 | 0.15 | 0.15 | 0.6 | 0.15 | 0.5908 | 0.112 | 0.141 |
| 0 | 0.3 | 0 | 0.15 | 0.15 | 0.15 | 0.3 | 0.1154 | 0.043 | 0.053 |
| 0 | 0.3 | 0 | 0.15 | 0.15 | 0.3 | 0.3 | 0.2802 | 0.117 | 0.131 |
| 0 | 0.6 | 0 | 0.15 | 0.15 | 0.15 | 0.6 | 0.0938 | 0.068 | 0.084 |
| 0 | 0.6 | 0 | 0.15 | 0.15 | 0.3 | 0.6 | 0.3281 | 0.306 | 0.349 |
| 0 | 0.6 | 0 | 0.15 | 0.15 | 0.6 | 0.6 | 0.7969 | 0.933 | 0.943 |
| 0 | 0.15 | 0 | 0.6 | 0.6 | 0.15 | 0.15 | 0.0614 | 0.009 | 0.013 |
| 0 | 0.15 | 0 | 0.6 | 0.6 | 0.3 | 0.15 | 0.2148 | 0.068 | 0.077 |
| 0 | 0.15 | 0 | 0.6 | 0.6 | 0.6 | 0.15 | 0.5217 | 0.134 | 0.162 |
| 0 | 0.3 | 0 | 0.6 | 0.6 | 0.15 | 0.3 | -0.0330 | 0.021 | 0.025 |
| 0 | 0.3 | 0 | 0.6 | 0.6 | 0.3 | 0.3 | 0.1319 | 0.081 | 0.090 |
| 0 | 0.6 | 0 | 0.6 | 0.6 | 0.15 | 0.6 | -0.3281 | 0.778 | 0.817 |
| 0 | 0.6 | 0 | 0.6 | 0.6 | 0.3 | 0.6 | -0.0938 | 0.120 | 0.156 |
| 0 | 0.6 | 0 | 0.6 | 0.6 | 0.6 | 0.6 | 0.375 | 0.881 | 0.901 |
| n=100 | | | | | | | | | |
| 0 | 0.15 | 0 | 0.15 | 0.15 | 0.15 | 0.15 | 0.1304 | 0.097 | 0.085 |
| 0 | 0.15 | 0 | 0.15 | 0.15 | 0.3 | 0.15 | 0.2839 | 0.291 | 0.287 |
| 0 | 0.15 | 0 | 0.15 | 0.15 | 0.6 | 0.15 | 0.5908 | 0.327 | 0.336 |
| 0 | 0.3 | 0 | 0.15 | 0.15 | 0.15 | 0.3 | 0.1154 | 0.163 | 0.162 |
| 0 | 0.3 | 0 | 0.15 | 0.15 | 0.3 | 0.3 | 0.2802 | 0.688 | 0.686 |
| 0 | 0.3 | 0 | 0.15 | 0.15 | 0.6 | 0.3 | 0.6099 | 0.864 | 0.875 |
| 0 | 0.6 | 0 | 0.15 | 0.15 | 0.15 | 0.6 | 0.0938 | 0.108 | 0.117 |
| 0 | 0.6 | 0 | 0.15 | 0.15 | 0.3 | 0.6 | 0.3281 | 0.793 | 0.802 |
| 0 | 0.6 | 0 | 0.15 | 0.15 | 0.6 | 0.6 | 0.7969 | 1.000 | 1.000 |
| 0 | 0.15 | 0 | 0.6 | 0.6 | 0.15 | 0.15 | 0.0614 | 0.068 | 0.060 |
| 0 | 0.15 | 0 | 0.6 | 0.6 | 0.3 | 0.15 | 0.2148 | 0.333 | 0.340 |
| 0 | 0.15 | 0 | 0.6 | 0.6 | 0.6 | 0.15 | 0.5217 | 0.301 | 0.311 |
| 0 | 0.3 | 0 | 0.6 | 0.6 | 0.15 | 0.3 | -0.0330 | 0.077 | 0.074 |
| 0 | 0.3 | 0 | 0.6 | 0.6 | 0.3 | 0.3 | 0.1319 | 0.567 | 0.564 |
| 0 | 0.3 | 0 | 0.6 | 0.6 | 0.6 | 0.3 | 0.4615 | 0.876 | 0.882 |
| 0 | 0.6 | 0 | 0.6 | 0.6 | 0.15 | 0.6 | -0.3281 | 1.000 | 1.000 |
| 0 | 0.6 | 0 | 0.6 | 0.6 | 0.3 | 0.6 | -0.0938 | 0.295 | 0.312 |
| 0 | 0.6 | 0 | 0.6 | 0.6 | 0.6 | 0.6 | 0.375 | 1.000 | 1.000 |

Table S6. Results from Alternative Conditions, Power for C as a confounder of M and Y

| **Conditions** | | | | | | | | **Power** | |
| --- | --- | --- | --- | --- | --- | --- | --- | --- | --- |
| $\boldsymbol{\rho}_{\boldsymbol{XC}}$ | $\boldsymbol{\rho}_{\boldsymbol{XM}}$ | $\boldsymbol{\rho}_{\boldsymbol{CM}}$ | $\boldsymbol{\rho}_{\boldsymbol{XY}}$ | $\boldsymbol{\rho}_{\boldsymbol{CY}}$ | $\boldsymbol{\rho}_{\boldsymbol{MY}}$ | $\boldsymbol{\alpha}_{\boldsymbol{1}}$ | $\boldsymbol{\gamma}_{\boldsymbol{3}}$ | **PSRM** | **IEFM** |
| n=30 | | | | | | | | | |
| 0 | 0.15 | 0.15 | 0.15 | 0.15 | 0.15 | 0.15 | 0.1099 | 0.007 | 0.008 |
| 0 | 0.15 | 0.15 | 0.15 | 0.15 | 0.3 | 0.15 | 0.2670 | 0.039 | 0.044 |
| 0 | 0.15 | 0.15 | 0.15 | 0.15 | 0.6 | 0.15 | 0.5812 | 0.126 | 0.152 |
| 0 | 0.3 | 0.15 | 0.15 | 0.15 | 0.15 | 0.3 | 0.0930 | 0.033 | 0.042 |
| 0 | 0.3 | 0.15 | 0.15 | 0.15 | 0.3 | 0.3 | 0.2620 | 0.099 | 0.109 |
| 0 | 0.3 | 0.15 | 0.15 | 0.15 | 0.6 | 0.3 | 0.6 | 0.352 | 0.398 |
| 0 | 0.6 | 0.15 | 0.15 | 0.15 | 0.15 | 0.6 | 0.0607 | 0.069 | 0.079 |
| 0 | 0.6 | 0.15 | 0.15 | 0.15 | 0.3 | 0.6 | 0.3036 | 0.233 | 0.269 |
| 0 | 0.6 | 0.15 | 0.15 | 0.15 | 0.6 | 0.6 | 0.7895 | 0.919 | 0.938 |
| 0 | 0.15 | 0.6 | 0.6 | 0.6 | 0.15 | 0.15 | -0.4858 | 0.174 | 0.209 |
| 0 | 0.15 | 0.6 | 0.6 | 0.6 | 0.3 | 0.15 | -0.2429 | 0.088 | 0.102 |
| 0 | 0.15 | 0.6 | 0.6 | 0.6 | 0.6 | 0.15 | 0.2429 | 0.081 | 0.090 |
| 0 | 0.3 | 0.6 | 0.6 | 0.6 | 0.15 | 0.3 | -0.7091 | 0.531 | 0.583 |
| 0 | 0.3 | 0.6 | 0.6 | 0.6 | 0.3 | 0.3 | -0.4364 | 0.529 | 0.578 |
| 0 | 0.3 | 0.6 | 0.6 | 0.6 | 0.6 | 0.3 | 0.1091 | 0.073 | 0.083 |
| n=100 | | | | | | | | | |
| 0 | 0.15 | 0.15 | 0.15 | 0.15 | 0.15 | 0.15 | 0.1099 | 0.065 | 0.057 |
| 0 | 0.15 | 0.15 | 0.15 | 0.15 | 0.3 | 0.15 | 0.2670 | 0.244 | 0.236 |
| 0 | 0.15 | 0.15 | 0.15 | 0.15 | 0.6 | 0.15 | 0.5812 | 0.313 | 0.327 |
| 0 | 0.3 | 0.15 | 0.15 | 0.15 | 0.15 | 0.3 | 0.0930 | 0.123 | 0.118 |
| 0 | 0.3 | 0.15 | 0.15 | 0.15 | 0.3 | 0.3 | 0.2620 | 0.607 | 0.606 |
| 0 | 0.3 | 0.15 | 0.15 | 0.15 | 0.6 | 0.3 | 0.6 | 0.878 | 0.889 |
| 0 | 0.6 | 0.15 | 0.15 | 0.15 | 0.15 | 0.6 | 0.0607 | 0.086 | 0.098 |
| 0 | 0.6 | 0.15 | 0.15 | 0.15 | 0.3 | 0.6 | 0.3036 | 0.678 | 0.691 |
| 0 | 0.6 | 0.15 | 0.15 | 0.15 | 0.6 | 0.6 | 0.7895 | 1.000 | 1.000 |
| 0 | 0.15 | 0.6 | 0.6 | 0.6 | 0.15 | 0.15 | -0.4858 | 0.431 | 0.455 |
| 0 | 0.15 | 0.6 | 0.6 | 0.6 | 0.3 | 0.15 | -0.2429 | 0.432 | 0.438 |
| 0 | 0.15 | 0.6 | 0.6 | 0.6 | 0.6 | 0.15 | 0.2429 | 0.445 | 0.448 |
| 0 | 0.3 | 0.6 | 0.6 | 0.6 | 0.15 | 0.3 | -0.7091 | 0.969 | 0.972 |
| 0 | 0.3 | 0.6 | 0.6 | 0.6 | 0.3 | 0.3 | -0.4364 | 0.970 | 0.972 |
| 0 | 0.3 | 0.6 | 0.6 | 0.6 | 0.6 | 0.3 | 0.1091 | 0.329 | 0.333 |
